# The Gut Microbiome as a Biomarker and Therapeutic Target in Hepatocellular Carcinoma

**DOI:** 10.3390/cancers15194875

**Published:** 2023-10-07

**Authors:** Betul Gok Yavuz, Saumil Datar, Shadi Chamseddine, Yehia I. Mohamed, Michael LaPelusa, Sunyoung S. Lee, Zishuo Ian Hu, Eugene J. Koay, Hop S. Tran Cao, Prasun Kumar Jalal, Carrie Daniel-MacDougall, Manal Hassan, Dan G. Duda, Hesham M. Amin, Ahmed O. Kaseb

**Affiliations:** 1 Department of Medicine, University of Missouri, St. Louis, MO 63121, USA; betulgokyavuz@gmail.com; 2 Department of Medicine, University of Texas at Houston, Houston, TX 77030, USA; saumil.datar@uth.tmc.edu; 3 Department of Gastrointestinal Medical Oncology, The University of Texas MD Anderson Cancer Center, Houston, TX 77030, USA; schamseddine@mdanderson.org (S.C.); yimohamed@mdanderson.org (Y.I.M.); sslee1@mdanderson.org (S.S.L.); zhu8@mdanderson.org (Z.I.H.); 4 Division of Cancer Medicine, The University of Texas MD Anderson Cancer Center, Houston, TX 77030, USA; mblapelusa@mdanderson.org; 5 Department of Radiation Oncology, The University of Texas MD Anderson Cancer Center, Houston, TX 77030, USA; ekoay@mdanderson.org; 6 Hepato-Pancreato-Biliary Section, Department of Surgical Oncology, The University of Texas MD Anderson Cancer Center, Houston, TX 77030, USA; hstran@mdanderson.org; 7 Division of Gastroenterology, Baylor College of Medicine, Houston, TX 77030, USA; jalal@bcm.edu; 8 Department of Epidemiology, The University of Texas MD Anderson Cancer Center, Houston, TX 77030, USA; cdaniel@mdanderson.org (C.D.-M.); mhassan@mdanderson.org (M.H.); 9 Steele Laboratories, Department of Radiation Oncology, Massachusetts General Hospital and Harvard Medical School, Boston, MA 02129, USA; duda@steele.mgh.harvard.edu; 10 Department of Hematopathology, The University of Texas MD Anderson Cancer Center, Houston, TX 77030, USA; hamin@mdanderson.org

**Keywords:** hepatocellular carcinoma, gut microbiome, therapeutic target, biomarkers

## Abstract

**Simple Summary:**

Hepatocellular carcinoma (HCC) is the most common type of liver cancer, accounting for approximately 85–90% of all cases of liver cancer worldwide. The gut microbiome can serve as a potential non-invasive biomarker for early HCC detection and may also impact the effectiveness of immunotherapy in cancer treatment. This review examines the gut microbiome’s role as a predictive and diagnostic marker for HCC and explores its potential as a novel therapeutic approach, particularly in the context of immunotherapy.

**Abstract:**

The microbiome is pivotal in maintaining health and influencing disease by modulating essential inflammatory and immune responses. Hepatocellular carcinoma (HCC), ranking as the third most common cause of cancer-related fatalities globally, is influenced by the gut microbiome through bidirectional interactions between the gut and liver, as evidenced in both mouse models and human studies. Consequently, biomarkers based on gut microbiota represent promising non-invasive tools for the early detection of HCC. There is a growing body of evidence suggesting that the composition of the gut microbiota may play a role in the efficacy of immunotherapy in different types of cancer; thus, it could be used as a predictive biomarker. In this review, we will dissect the gut microbiome’s role as a potential predictive and diagnostic marker in HCC and evaluate the latest progress in leveraging the gut microbiome as a novel therapeutic avenue for HCC patients, with a special emphasis on immunotherapy.

## 1. Introduction

Hepatocellular carcinoma (HCC) is one of the most common types of liver cancer, accounting for a significant number of cancer-related deaths worldwide [1,2,3,4]. The insidious progression of this cancer, combined with the lack of effective biomarkers for its detection, make it difficult to diagnose at an early stage when treatment is most effective. Surgical resection remains the only curative approach for HCC, but it is only available for patients with localized tumors [5]. Therefore, there is a need to identify new biomarkers and therapeutic targets to improve the diagnosis and treatment of HCC.

Recent research emphasizes the role of the gut microbiome in modulating the immune system and its involvement in various disease states [6]. The gut microbiome has been shown to play a critical role in the development of immune tolerance and response, which is particularly relevant in the context of cancer [7]. Emerging evidence suggests that the gut microbiome can also impact HCC development and progression through gut–liver bidirectional interactions.

Overall, these studies suggest that the gut microbiome could serve as a biomarker and therapeutic target for HCC. The identification of specific microbial signatures and the use of interventions to restore gut microbiome balance could provide a new avenue for the prevention and treatment of HCC. In this review, we discussed the importance of the gut microbiome both as a diagnostic or predictive biomarker and as a therapeutic target through the modulation of the immune system.

## 2. Discussion

### Mechanisms by Which the Gut Microbiota Mediates the Development of Hepatocellular Carcinoma

Dysbiosis, an imbalance in the gut microbiome, coupled with intestinal permeability, wherein harmful substances and bacteria can traverse from the gut into the bloodstream, have been identified as mechanisms that have been shown to contribute to the development of HCC [8]. The liver and gastrointestinal tract maintain bidirectional communication via the gut–liver axis, primarily through the portal vein. This vein channels gut-derived microbiota, their products, and metabolites to the liver. While a healthy gut barrier ensures minimal entry of harmful compounds from the abundant bacterial species in the colon, factors like fatty liver disease, liver fibrosis, alcohol consumption, antibiotics, and diet can cause dysbiosis. This imbalance, often resulting from a compromised gut barrier function, makes the intestinal barrier more permeable. This increased “leakiness” allows more microbiota-related patterns and metabolites to access the liver, exacerbating chronic liver disease (CLD) progression and heightening liver cancer risk while altering both gut and liver immune functions [8,9,10].

Beharry et al. showed that the microbiota in NAFLD-HCC plays a role in inducing an immunosuppressive response characterized by the expansion of IL-10+ Tregs, decreased pro-inflammatory cytokine production, such as IL-2 and IL-12, and attenuation of cytotoxic CD8+ T cells. This immunosuppressive environment may contribute to the progression and development of NAFLD-HCC [11]. Another study on mice showed that dysbiosis heightened infiltration of myeloid-derived suppressor cells (MDSCs) to the liver, fostering liver carcinogenesis with an associated decrease in *Akkermansia muciniphila* in the gut [12]. Furthermore, the analysis of fecal and serum metabolomics revealed elevated concentrations of short-chain fatty acids (SCFAs) and their intermediates in NAFLD-HCC subjects when compared to those with NAFLD-cirrhosis and non-NAFLD controls [11].

The bacterial fermentation of non-digestible carbohydrates produces SCFAs, like butyrate and propionate, which have been linked to the regulation of CD4+ and CD8+ T cells and the reduction in inflammation [13,14]. Along these lines, butyrate-producing bacterial genera, including *Ruminococcus*, *Oscillibacter*, *Faecalibacterium*, *Clostridium IV*, and *Coprococcus*, were found to be decreased while LPS-producing bacteria, including *Klebsiella* and *Haemophilus*, were found to be increased in patients with HCC compared to healthy subjects [15]. Secondary bile acids, which are produced by the gut microbiome from primary bile acids and reabsorbed in the intestine, have been shown to promote hepatic inflammation and hepatocarcinogenesis [10,16,17].

## 3. Gut Microbiome as a Diagnostic Biomarker to Detect HCC

Most patients with HCC are diagnosed at advanced stages. The gut microbiome offers potential as an early HCC detection biomarker (Table 1). Previous analyses have shown that microbial diversity was significantly decreased in liver cirrhosis compared to healthy controls but increased in HCC compared to cirrhosis [15,18]. The findings suggested that the presence of liver cirrhosis may be the main cause of gut microbial dysbiosis in HCC patients. Another study by Lapidot et al., revealed that a random forest classifier was used to distinguish patients with HCC-cirrhosis from healthy controls based on fecal microbiome composition, resulting in an overall accuracy of 82% and an area under the receiver operating characteristic curve (AUC) value of 0.9. Significant differences in bacterial composition were observed between patients with HCC-cirrhosis and those with cirrhosis only, with an overrepresentation of *Clostridium* and *CF231* (belonging to the family of Paraprevotellaceae) and reduced *Alphaproteobacteria* abundance compared to cirrhotic patients without HCC [19].

Ponziani et al. showed that patients with NAFLD-induced cirrhosis and HCC had increased gut permeability, leading to heightened levels of circulating fecal calprotectin, inflammatory cytokines, and activated monocytes when compared to those with NAFLD-induced cirrhosis without HCC. Moreover, HCC presence correlated with increased *Bacteroides* and *Ruminococcaceae*, while gut-protective bacteria such as *Bifidobacterium* diminished [20]. In another study, comparing the HBV-associated HCC group to healthy controls, *Bacteroides*, *Lachnospiracea incertae sedis*, and *Clostridium XIVa* were found to be enriched in the HCC group. Within the HCC group, these genera were significantly enriched in the non-small HCC subgroup compared to the small HCC subgroup, which was defined based on the tumor burden [21].

Shotgun metagenomic sequencing of fecal samples confirmed dysbiosis in both the NAFLD-HCC and NAFLD-cirrhosis groups compared to non-NAFLD controls [11]. Subjects with NAFLD-HCC and NAFLD-cirrhosis had reduced microbial diversity compared to non-NAFLD controls. At the phylum level, NAFLD-HCC was characterized by an expansion of *Proteobacteria* compared to non-NAFLD controls. At the family level, NAFLD-HCC was characterized by an expansion of *Enterobacteriaceae* and a reduction in *Oscillospiraceae* and *Erysipelotrichaceae* compared to non-NAFLD controls [11]. In a study with a total of 90 subjects, *Bacteroides caecimuris* and *Veillonella parvula* were found to be significantly enriched in NAFLD-HCC compared to NAFLD-cirrhosis and non-NAFLD controls [11].

In a study where the alteration of gut microbiota was investigated in healthy controls, HBV-related HCC (B-HCC) patients and non-HBV, non-HCV (NBNC)-related HCC (NBNC-HCC) patients revealed distinct differences in bacterial composition among the three groups. B-HCC patients had a much higher species richness of fecal microbiota than the other two groups. It is interesting to note that the feces of NBNC-HCC patients had lower concentrations of *Faecalibacterium*, *Ruminococcus*, and *Ruminoclostridium*, which were associated with short-chain fatty acids, and higher concentrations of potentially pro-inflammatory bacteria, including *Escherichia-Shigella* and *Enterococcus* [22].

Recently, bacterial colonization, termed cancer microbiota, were found in various tumor tissues, including those previously thought to be sterile, like breast and pancreatic cancers, although replication of the results has been challenging [26,27]. Given the liver’s anatomical link to the intestines through the portal vein and the migration of gut bacteria to the liver in patients with chronic liver disease, it is possible that HCC may also harbor cancer-associated microbiota. A recent study suggested that liver cancers, both primary and metastatic, have distinct microbial compositions compared to non-tumor regions [28]. Huang et al. investigated the presence of viable bacteria in liver cancer by performing cultures from fresh HCC tissues. Positive cultures yielded visible colonies, including *Staphylococcus aureus*, *Rothia*, *Bacillaceae*, and *Corynebacterium* species. This suggested that viable and infectious bacteria were present in HCC tissues. Moreover, they showed that the microbiota of HCCs and peritumor tissues had higher alpha diversity, which is defined as a measure of microbiome diversity within a single sample, compared to normal liver tissues with enrichment of some species. The study further explored the potential of intratumoral microbial signatures as diagnostic or prognostic biomarkers for HCC. The models achieved high accuracy in distinguishing HCC subjects from normal subjects in both the training and validation cohorts. The top five class species, including *Bacilli*, *Acidobacteriae*, *Parcubacteria*, *Saccharimonadia*, and *Gammaproteobacteria*, were identified as important features for HCC prediction [24]. Nonetheless, the existence of microbiota in liver cancer and its clinical significance are yet to be conclusively established.

Circulating microbial signature is another emerging subject in the cancer field and is thought to be partially derived directly from the gut via bacterial translocation [29]. Cho et al. investigated the relationship between hepatocellular carcinoma and alterations in the composition of the circulating microbiota. Blood microbial diversity in HCC was notably lower than in cirrhosis and controls. Several bacterial taxa showed significant variations in abundance associated with HCC, indicating a distinct blood microbiome signature for HCC. They identified a five-microbial-gene-markers-based model that was able to accurately discriminate HCC from controls, with an AUC of 0.875, suggesting a potential for the blood-microbiome-based signature as a diagnostic tool for HCC [23]. Further studies are needed to identify circulating HCC microbial signatures and concordance with the gut microbiome.

## 4. Gut Microbiome as a Marker in Immunotherapy Response

Immune checkpoint inhibitors (ICIs) have recently been approved as frontline or secondary treatments for hepatocellular carcinoma (HCC), exhibiting benefits in patient survival [30]. The most notable advancement is the emergence of atezolizumab, an anti-PD-L1 antibody, combined with bevacizumab, an anti-VEGF antibody, as the new preferred standard first-line therapy for advanced HCC after atezolizumab plus bevacizumab was shown to improve survival against sorafenib [31]. Despite these strides in HCC treatment using ICIs, the current strategies remain non-curative in the advanced and metastatic setting and roughly a quarter of patients encounter severe (grade 3–4) immune-related side effects [32,33]. Consequently, identifying predictive biomarkers of clinical response to immunotherapy holds substantial potential to refine patient selection and enhance treatment outcomes.

There is a growing body of evidence suggesting that the composition of the gut microbiota may play a role in the efficacy of immunotherapy in different types of cancer (Table 2). Ll et al. showed that high abundance of *Faecalibacterium* in patients was associated with significantly prolonged progression-free survival (PFS) compared to those with low abundance. Conversely, patients with high abundance of *Bacteroidales* had a shortened PFS compared to those with low abundance [34]. They further explored the differences between responders and non-responders to ICIs therapy, which identified differentially abundant bacteria in the fecal microbiome, with *Clostridiales/Ruminococcaceae* enriched in the response group and *Bacteroidales* enriched in the non-response group [34]. Another study involving eight patients with HCC who received anti-PD-1 treatment found that there was no significant gut microbiome dysbiosis at baseline between responders and non-responders. However, dynamic analysis during anti-PD-1 immunotherapy showed that those who responded to the treatment had fecal samples with higher taxonomic diversity and more gene counts across twenty different species, including *Akkermansia* and *Ruminococcaceae*, compared to non-responders who were enriched for *Proteobacteria*. This difference was apparent as early as six weeks after treatment initiation, indicating that the composition of the gut microbiota could potentially serve as an early predictor of treatment outcome [35]. Another study involving seventy-four patients with advanced-stage gastrointestinal (GI) cancer who received anti-PD-1/PD-L1 treatment found that patients who exhibited a higher prevalence of *Prevotella* and a reduced presence of *Bacteroides* demonstrated a higher likelihood of achieving progression-free survival (PFS) within 12 weeks of initiating treatment. In the GI cancer group, *Akkermansia* was linked to a positive response to anti-PD-1/PD-L1 therapy, whereas *Lactobacillus* was not [36]. Pathway analysis indicated variations in metabolic and biological processes between responders and non-responders. Those who responded showed a higher prevalence in pathways linked to SCFA fermentation, unsaturated fatty acid creation, and the biosynthesis of vitamins and starch. In contrast, non-responders displayed a dominant presence in pathways associated with lipopolysaccharide creation, sugar breakdown, and the formation of amino acids [36].

The gut microbiome was shown to change dynamically during immunotherapy, and certain species were found to be associated with treatment response [37,40]. The study investigating the effects of gut microbiota on the efficacy of nivolumab in eight adult HCC patients who received nivolumab as second- or third-line treatment after sorafenib failure showed that responders to nivolumab therapy exhibited a higher Shannon index and alpha diversity indices than non-responders, indicating greater species richness in the former group [37]. There were certain bacterial species that were more abundant in non-responders (*Ruminococcus gnavus*) and responders (e.g., *Clostridia*, *Prevotella 9*, *Rikenellaceae*, *Alistipes*, *the Christensenellaceae R-7 group*, *Dialister*, etc.). Furthermore, responders tended to have a more favorable *Firmicutes/Bacteroidetes* ratio and a higher *Prevotella* species to *Bacteroides* species (P/B) ratio, and they were more likely to have *Akkermansia* species in their gut [37].

Fecal calprotectin is a marker showing intestinal inflammation and found to be changing through ICI treatment [40]. HCC patients who achieved disease control with tremelimumab plus durvalumab had lower fecal calprotectin concentrations and pretreatment abundance of *Akkermansia* compared to non-responders and showed an inverse trend compared to the ratio of *Akkermansia* to Enterobacteriaceae (AE ratio), used as a marker of dysbiosis [40]. This confirms the link between *Akkermansia* and an improved response observed in patients with various solid tumors undergoing ICI treatment, aligning with the anti-inflammatory attributes of *Akkermansia* [41]. Contrarily, another study demonstrated no notable differences in the baseline gut microbiome’s alpha diversity, richness, and composition between responders and non-responders among HCC patients receiving ICIs. The main microbiome features remained unaffected by immunotherapy, highlighting significant variations between different studies, likely emerging from experimental workflow, starting from stool sampling to bioinformatic analyses [42].

Circulating metabolites that are produced by the microbiome have been shown to carry value as a predictive marker. A prospective clinical study led by Dr. Wu et al., investigated the role of the gut microbiome and its metabolites, including galactaric acid, 13-L-hydroperoxylinoleic acid, formononetin, alpha-D-glucose, and arachidonic acid, in predicting response to immunotherapy. They showed that, in contrast to the gut microbiome classifier, the blood-metabolites-based classifier was better able to identify HCC patients who benefited from immunotherapy at baseline (AUC 0.793, 95% CI: 0.632–0.954) [38]. In another recent study, two key gut microbiota (KGM; Odoribacter splanchnicus and Ruminococcus bicirculans) and five key serum metabolites (KSM; ouabain, taurochenodeoxycholic acid, glycochenodeoxycholate, theophylline, and xanthine) were found to be associated with HCC compared to non-HCC patients. Similar to the study by Wu et al., the metabolite-based panel was found to outperform the gut-microbiome-based panel in differentiating HCC from non-HCC. However, it performed poorly at differentiating HCC from cirrhosis, with an AUC value of less than 0.7. Importantly, combining both panels improved AUC for HCC versus cirrhosis (AUC > 0.7), suggesting the superior predictive value of the combined metabolite- and microbiome-based panels [25]. In another prospective study with patients receiving ICI treatment for unresectable HCC, it was observed that the baseline gut microbiota were distinct between ICI responders and non-responders [39]. *Prevotella 9* was more abundant in patients with progressive disease, while *Lachnoclostridium*, *Lachnospiraceae*, and *Veillonella* were more prevalent in patients with objective response. The study also examined the association of gut microbial metabolites with treatment response. Patients with objective response showed a marked increase in secondary bile acids in their feces, including UDCA, tauro-UDCA, UCA, and MDCA, but no significant difference was observed in the fecal concentration of short-chain fatty acids [39].

## 5. Gut Microbiome as Therapeutic Target

The concept of microbiota-based therapies, including probiotics, prebiotics, antibiotics, and fecal microbiota transplantation (FMT), is not new and has shown promise in treating several conditions, from metabolic disorders to autoimmune diseases. Modulating the gut microbiome could offer a novel approach for both prevention and treatment of HCC (Table 3).

Animal studies have shown that targeting gut microbiota could reduce hepatocarcinogenesis by reducing chronic inflammation via decreasing translocating inflammatory mediators such as LPS [43,44]. Rifaximin is a routinely used antibiotic for patients with cirrhosis to prevent hepatic encephalopathy. It has been shown that rifaximin use is associated with reduced complications in cirrhotic patients, including hepatic encephalopathy, variceal bleeding, and spontaneous bacterial peritonitis [45,46]. However, its effect on the development of HCC has yet to be explored.

## 6. Antibiotics

The use of antibiotics, known disruptors of gut microbiota, could potentially influence outcomes in patients receiving ICIs. Several meta-analyses have reported worse outcomes in patients with lung, melanoma, bladder, and kidney cancers treated with ICIs who were concomitantly administered antibiotics, possibly altering the balance between favorable and unfavorable bacterial species [47,48].

The studies investigating the impact of antibiotics on ICI treatment in HCC have yielded contradictory findings. The concurrent antibiotic use within 30 days of immunotherapy treatment in patients with advanced HCC was associated with significant increases in cancer-related and all-cause mortality, with the effects being more pronounced with anti-aerobic than anti-anaerobic activity in a territory-wide retrospective cohort study with 395 HCC patients [49]. Similarly, other retrospective studies showed that patients with HCC receiving ICIs have worse survival outcomes if they also received antibiotics [50,51]. One possible explanation for this would be that antibiotics could decrease microbial diversity, which was associated with ICIs response [35]. Interestingly, another study with an international cohort of 450 patients showed that antibiotic exposure within 30 days before or after the start of ICI treatment was associated with longer PFS, regardless of disease- and treatment-related features [52]. The authors concluded that this favorable response to ICI with antibiotic use could be attributed to the mitigation of gut dysbiosis, subsequently leading to a decrease in immunosuppressive interactions [52]. The recent meta-analysis led by Zhang et al. showed that antibiotic use did not have an impact on the OS and PFS in HCC patients treated with ICIs [53]. The discrepancy between the results could be partly explained by the lack of subgroup analysis based on the type of antibiotic used, route of administration, duration of use, etc. Overall, antibiotic use and ICI interaction might be more complex in the setting of HCC than in other cancer types since HCC develops in the context of cirrhosis, an already immunosuppressive condition characterized by an imbalanced gut microbiome, and we need more standardized, large-scale research to clarify the role of antibiotics in ICI outcomes for HCC.

Ma et al. showed a connection between the gut microbiome, bile acids, and liver cancer [10]. The study showed that certain Gram-positive bacteria in the gut, like Clostridium, modified bile acids, influencing the production of chemokine CXCL16 in liver cells. This stimulated an influx of natural killer T (NKT) cells to the liver, which were key in controlling tumor growth. By manipulating the gut bacteria and altering the bile acid levels, it was possible to modulate NKT cell recruitment and tumor growth. Importantly, using antibiotic treatment with vancomycin to eliminate Gram-positive bacteria, which facilitate the conversion from primary to secondary bile acid, effectively led to an increase in NKT cells in the liver and a reduction in liver tumor growth [10]. These findings prompted researchers to test whether vancomycin improves the response to nivolumab in patients with refractory primary HCC or liver-dominant metastatic cancer in a clinical trial (NCT03785210), which is still ongoing [54].

Another retrospective study investigating the role of antibiotics on OS and PFS in patients with advanced HCC treated with sorafenib showed that antibiotic use was independently associated with worse outcomes in HCC patients treated with sorafenib. This association is possibly attributed to alterations in gut microbiome composition, which influences the enterohepatic recycling of sorafenib, affecting its metabolism and the occurrence of side effects in patients with HCC [55,56].

## 7. Probiotics and Prebiotics

Probiotics are live microorganisms and have been studied in different disease settings including cancer. Probiotic administration can modulate the host’s gut microbiota by increasing the proliferation of beneficial microbes while suppressing the growth of microbes linked to HCC-induced dysbiosis, thereby averting hepatic inflammation caused by pathogen-associated molecular patterns (PAMPs) [57,58]. Animal studies showed that probiotic administration has been shown to decrease HCC progression by reducing the Th17 polarization and promoting the differentiation of anti-inflammatory Treg/Tr1 cells in the gut [59]. The retrospective study of 1267 patients with hepatitis-B-related cirrhosis investigated the potential link between probiotics and HCC risk during antiviral therapy. The study found that probiotics were an independent protective factor against HCC and a significantly lower incidence of HCC was found among probiotic users, with a clear dose–response pattern observed, suggesting that adjuvant probiotic therapy could reduce the risk of HCC in these patients [60].

Administration of probiotic formulations with certain species has been intended to increase the immunotherapy effect in different cancer settings, including melanoma and RCC [61,62,63]. A clinical trial involving 46 liver cancer patients is currently recruiting patients to assess the impact of administering the *Lactobacillus rhamnosus* Probio-M9 probiotic to enhance the response to anti-PD-1 therapy (NCT05032014) [64].

Prebiotics are a group of food ingredients selectively utilized by the gut microbiome. Their effects largely stem from the generation of metabolites when prebiotics are fermented by specific genera/species in the gut microbiota, including *Lactobacilli* and *Bifidobacteria* [65]. Diet and prebiotics can quickly impact the human gut microbiome [66]. Dietary fiber and probiotic intake have been shown to be associated with improved progression-free survival in patients with melanoma receiving immunotherapy, possibly increasing cytotoxic T cells in the tumor microenvironment [67]. Human studies that investigate the effect of prebiotics on HCC development and treatment are lacking, although animal studies have shown positive results [68,69].

## 8. Fecal Microbiota Transplantation

FMT involves transferring the fecal matter from a donor into the gastrointestinal tract of a recipient. It has emerged as a potential therapeutic approach across different disease entities. FMT is currently approved only for antibiotic-resistant clostridium difficile infection, with resolution in 80% to 90% of patients [70]. Baruch et al. performed the first phase 1 clinical trial of FMT from anti-PD1 therapy-responsive donors on melanoma and showed that FMT was associated with a favorable response by increasing intratumoral immune activity [71]. A prospective single-arm clinical trial (NCT04264975) is investigating the impact of FMT on patients with advanced solid cancer, including HCC resistant to anti-PD-L1 inhibitors. The preliminary results indicated that, among thirteen patients, one showed a partial response and five exhibited stable disease after FMT. The clinical response was associated with an increase in cytotoxic T cells in both the blood and tumor environment, immune cytokines, and an enhanced presence of a novel species closely related to *Prevotella* sp. *Marseille-P4119*. This suggests that FMT containing beneficial microbiota might help overcome resistance to immunotherapy by altering the tumor microenvironment [72]. A phase II trial (NCT05690048), named as FLORA (Fecal Microbiota Transfer in Liver Cancer to Overcome Resistance to Atezolizumab/Bevacizumab), will assess the safety and immunogenicity of fecal microbiota transfer in combination with standard-of-care immunotherapy in advanced hepatocellular carcinoma [73]. Similarly, another phase II trial (NCT05750030) will evaluate the safety, feasibility, and efficacy of FMT from patients with HCC who responded to PD-L1-based immunotherapy to patients with HCC who failed to respond to atezolizumab/bevacizumab [74].

FMT has a promising role in the treatment of colitis among patients receiving immunotherapy. The first study to evaluate FMT as a first-line treatment for immune-mediated colitis due to immunotherapy showed that, of the seven patients enrolled, 71.4% experienced symptom improvement within a day after FMT, and 85.7% were able to resume cancer treatment, indicating that FMT may be a safe and effective steroid-sparing alternative for immunotherapy-mediated colitis treatment [75]. FMT was also shown to be effective in treatment of refractory immune-related colitis as salvage therapy [76]. FMT might mitigate ICI-related colitis by reconstitution of the gut microbiome, increasing the number of regulatory T cells in the gut mucosa [77].

## 9. Conclusions: Future Outlook

The bidirectional connection between gut microbiota and the liver plays a pivotal role in the mechanisms underlying liver diseases including HCC. There is growing evidence pointing to the influence of the gut microbiota in the onset and progression of HCC. Moreover, the gut microbiota presents potential as an early diagnostic and predictive marker for therapy for HCC. Consequently, adjusting the gut microbiota could emerge as a novel strategy for HCC prevention or treatment.

While the gut microbiome presents a promising avenue in HCC research, considerable challenges lie ahead. Firstly, most of the knowledge on this subject is obtained from mice, which are not identical to humans in gut composition [78]. The differences in microbial composition between species can translate to variations in the disease process and treatment response. Lack of standardization in sample collection, microbiome analysis methodologies, and interpretative strategies poses a significant challenge, leading to disparities in outcomes. As the field progresses, there is a pressing need to establish consistent standards, ensuring that the findings are reliable, reproducible, and comparable across studies. Furthermore, we need more prospective, controlled, and randomized studies to better understand and validate the role of gut microbiome in HCC. Lastly, it is essential to consider the role of microbiota beyond the gut. Intratumoral microbiota has been shown to be present in a variety of tumor tissues and exhibit an association with the onset and progression of cancer, as well as the effectiveness of therapeutic interventions. Investigating these diverse microbial niches, including tumor tissue, may offer a more holistic understanding of the intricate relationship between microbes and HCC.

## Figures and Tables

**Table 1:** The studies investigating the relationship between gut microbiome and HCC.

Study	(N)	Etiology	Method	Bacteria Associated with HCC Compared to Other Groups	Ref.
Ren et al.	HCC (150), 40 cirrhosis (40), healthy controls (131)	HBV	16S rRNA sequencing	HCC: *Klebsiella* and *Haemophilus*, (LPS-producing bacteria) Control: *Ruminococcus*, *Oscillibacter*, *Faecalibacterium*, *Clostridium IV*, and *Coprococcus*, (butyrate-producing bacteria)	[15]
Ponziani et al.	NAFLD-related cirrhosis and HCC (21), NAFLD-related cirrhosis without HCC (20), healthy controls (20)	NAFLD	16S rRNA sequencing	HCC: *Bacteroidetes* at the phylum level, *Bacteroidaceae*, *Streptococcaceae*, *Enterococcaceae*, and *Gemellaceae* at the family level; and *Phascolarctobacterium*, *Enterococcus*, *Streptococcus*, *Gemella*, and *Bilophila* at the genus level	[20]
Behary et al.	NAFLD-HCC (32), NAFLD-cirrhosis (28), non-NAFLD controls (30)	NAFLD	Shotgun metagenomic sequencing	NAFLD-HCC (vs. non-NAFLD controls): Abundance in *Enterobacteriaceae* and a reduction in *Oscillospiraceae* and *Erysipelotrichaceae*. NAFLD-cirrhosis (vs. non-NAFLD controls): an expansion of *Eubacteriaceae* and a reduction in several *Bacteroidetes* families.	[11]
Huang et al.	HCC (113), healthy controls (100)	HBV	16S rRNA sequencing	HCC: *Bacteroides*, *Lachnospiracea* incertae sedis, and *Clostridium XIVa*	[21]
Lapidot et al.	cirrhosis (38), HCC-cirrhosis (30), age- and BMI-matched healthy controls (27)	NAFLD and HCV	16S rRNA sequencing	HCC-cirrhosis: Redcution in butyrate-producing bacteria *Ruminococcaceae*, *Butyricicoccus*, and *Lachnospiraceae* and abundance of genera *Lachnospira*, *Anaerostipes*, and *Christensenella*.	[19]
Liu et al.	Healthy controls (3), HBV-HCC (35), non-HBV non-HCV-related HCC (NBNC-HCC) (22)	HBV-related HCC and non-HBV non-HCV (NBNC) HCC	16S rRNA sequencing	B-HCC (vs. healthy controls): *Prevotella*, *Phascolarctobacterium*, and *Anaerotruncus* NBNC-HCC (vs. healthy controls): *Escherichia-Shigella* and *Enterococcus*	[22]
Cho et al.	HCC (158), cirrhosis (166), healthy controls (402)	Viral and non-viral	Metagenomic sequencing	HCC: Abundance of *Staphylococcus*, *Acinetobacter*, *Klebsiella*, *and Trabulsiella*, reduction in *Pseudomonas*, *Streptococcus*, and *Bifidobacterium*	[23]
Huang et al.	28 normal liver, 64 peritumoral, and 64 HCC tissues	Viral and non-viral	16S rRNA sequencing	HCC: *Patescibacteria*, *Proteobacteria*, *Bacteroidota*, *Firmicutes*, and *Actinobacteriota* at the phylum level. HCC and peritumoral tissues: *Proteobacteria*, *Firmicutes*, and *Actinobacteriota* at the phylum level, and classes of *Bacilli* and *Actinobacteria*	[24]
Li et al.	HCC (68), cirrhosis (33), healthy individuals (34)	Viral and non-viral	Metagenome sequencing LC-MS for metabolite	HCC: *Odoribacter splanchnicus* and *Ruminococcus bicirculans*) and five key metabolites (ouabain, taurochenodeoxycholic acid, glycochenodeoxycholate, theophylline, and xanthine)	[25]

**Table 2:** The studies investigating the relationship between gut microbiome and immunotherapy treatment.

Study	N	Method	Bacteria Associated with Response (R) or Non-Response (NR)	Immunotherapy	Ref.
Ll et al.	65	16S rRNA sequencing	R: *Faecalibacterium* and *Bacteroidales*,	ICIs (unspecified)	[34]
Zheng et al.	8	metagenomic sequencing	R: *Akkermansia* and *Ruminococcaceae* NR: Proteobacteria	Anti-PD-1 treatment	[35]
Chung et al.	8	16S rRNA sequencing	R: *Dialister pneumosintes*, *Escherichia coli*, *Lactobacillus reteri*, *Streptococcus mutans*, *Enterococcus faecium*, *Streptococcus gordonii*, *Veillonella atypica*, *Granulicatella* sp., and *Trchuris trichiura* for the non-responders; *Citrobacter freundii*, *Azospirillum* sp., and *Enterococcus durans* R: A higher *Prevotella* species to *Bacteroides* species (P/B) ratio R: Akkermansia species	nivolumab	[37]
Wu et al.	61 patients	16S rRNA sequencing	R: *Faecalibacterium*, *Blautia*, *Lachnospiracea incertae Sedis*, *Megamonas*, *Ruminococcus*, *Coprococcus*, *Dorea*, and *Haemophilus* NR: *Atopobium*, *Leptotrichia*, *Campylobacter*, *Allisonella*, *Methanobrevibacter*, *Parabacteroides*, *Bifidobacterium*, and *Lactobacillus*	Anti-PD-1 treatment	[38]
Lee et al.	41 patients	16S rRNA sequencing	R: *Lachnoclostridium*, *Lachnospiraceae*, and *Veillonella* NR: *Prevotella 9*	nivolumab and pembrolizumab	[39]
Peng et al.	85 patients with gastrointestinal cancers	16S rRNA sequencing	R: *Ruminococcaceae*, *Prevotella*, and *Lachnospiraceae* NR: *Bacteroides*, *Catenibacterium*, *Ruminococcaceae_NK4A214_group*.	anti-PD-1/PD-L1 treatment	[36]

**Table 3:** Clinical trials of gut-microbiome-based therapies in HCC.

Clinical Trial	Official Title	Intervention	Research Purpose	Primary Outcome	Status
NCT03785210	Phase II Study of Nivolumab (Anti-PD1), Tadalafil and Oral Vancomycin in Patients With Refractory Primary Hepatocellular Carcinoma or Liver Dominant Metastatic Cancer From Colorectal or Pancreatic Cancers	Vancomycin	To investigate if nivolumab given with tadalafil and vancomycin causes liver cancer to shrink.	Best overall response	Completed
NCT05032014	Probiotics Enhance the Treatment of PD-1 Inhibitors in Patients With Liver Cancer	Probio-M9	To assess whether probiotics can improve the efficacy of ICI	Proportion of patients whose tumor volume shrinks to a predetermined value and maintains the minimum time limit	Recruiting
NCT04264975	Utilization of Microbiome as Biomarkers and Therapeutics in Immuno-Oncology	FMT	To evaluate whether the fecal microbiota transplantation (FMT) could help overcome resistance in pts with advanced solid cancer refractory to anti-PD-(L)1 inhibitors	Overall response rate	Unknown
NCT05690048	Fecal Microbiota Transfer in Liver Cancer to Overcome Resistance to Atezolizumab/Bevacizumab	FMT	To assess safety and immunogenicity of fecal microbiota transfer in combination with standard of care immunotherapy in advanced hepatocellular carcinoma	Differential tumoral CD8 T-cell infiltration Adverse event documentation of FMT in advanced HCC	Not yet recruiting
NCT05750030	Fecal Microbiota Transplant (FMT) Combined With Atezolizumab Plus Bevacizumab in Patients With HepatoCellular Carcinoma Who Failed to Respond to Prior Immunotherapy—the FAB-HCC Pilot Study	FMT	To assess the safety of FMT combined with atezolizumab plus bevacizumab, as measured by incidence and severity of treatment-related adverse events	Safety of atezolizumab/bevacizumab in combination with FMT, measured by incidence and severity of treatment-related adverse events, determined according to National Cancer Institute (NCI) Common Terminology Criteria for Adverse Events (CTCAE)	Not yet recruiting

## Data Availability

The data presented in this study are available in this article.
